# Understanding the Benefits of CO_2_ Laser Treatment for Vulvovaginal Atrophy

**DOI:** 10.3390/medicina60071059

**Published:** 2024-06-27

**Authors:** Svetlana Jankovic, Marija Rovcanin, Ana Tomic, Aleksandar Jurisic, Zagorka Milovanovic, Milena Zamurovic

**Affiliations:** 1 Clinic for Gynecology and Obstetrics, Narodni Front, Kraljice Natalije 62, 11000 Belgrade, Serbia; rovcanin.marija@gakfront.org (M.R.); ajurisic@jurisicordinacija.rs (A.J.); zaga_mil@yahoo.co.uk (Z.M.); mzamurovic@gmail.com (M.Z.); 2 Faculty of Medicine, University of Belgrade, Dr Subotica Starijeg 8, 11000 Belgrade, Serbia; 3 Center for Radiology and Magnetic Resonance Imaging, University Clinical Center of Serbia, 11000 Belgrade, Serbia; anatomic9977@gmail.com

**Keywords:** vulvovaginal atrophy, female sexual health, menopause, fractional CO_2_ laser

## Abstract

*Background and Objectives:* Postmenopausal vaginal discomfort is often attributed to vulvovaginal atrophy (VVA). Women with VVA experience symptoms such as vaginal dryness, itching, burning, irritation, and dyspareunia. *Materials and Methods:* This pilot study was conducted to assess the effects of a micro-ablative fractional CO_2_ laser on the clinical symptoms of VVA, as well as concordant sexual function. The severity of VVA symptoms was evaluated by a visual analogue scale (VAS), while the condition of the vaginal mucosa was evaluated using the Vaginal Health Index Score (VHSI). Sexual function was evaluated using the Female Sexual Function Index (FSFI) Questionnaire. *Results:* Our cohort included 84 sexually active postmenopausal women with bothersome VVA, leading to sexual health complaints. The mean age of the participants in our study was 55.2 ± 5.4 years, with an average postmenopausal period of 6 ± 4.8 years. The age of our patients and the length of their postmenopausal period exhibited a significant negative correlation with VHSI scores, while a longer postmenopausal period was associated with increased severity of vaginal dryness and dyspareunia. Baseline VHSI values showed that 65% of patients had atrophic vaginitis with pronounced VVA symptoms (70.2% experienced vaginal itching, 73.8% reported vaginal burning, 95.3% had vaginal dryness, and 86.1% suffered from dyspareunia). Lower VHSI values significantly correlated with lower FSFI scores, while more severe VVA symptoms scores correlated with lower FSFI scores. VVA symptoms were significantly less severe after treatment. VHIS regained high non-atrophic values in 98.8% of patients post-treatment (*p* < 0.001). FSFI total and domain scores were significantly higher after treatment (*p* < 0.001). *Conclusions:* Our study revealed that fractional CO_2_ laser is a useful treatment option to alleviate VVA symptoms and improve vaginal health and sexual functioning in postmenopausal women.

## 1. Introduction

Vulvovaginal atrophy (VVA), in literature, often named atrophic vaginitis, is a common disorder that is especially prevalent in postmenopausal women [1]. By new terminology, VVA is a component of a collection of various subjective symptoms and objective examination findings named genitourinary syndrome of menopause (GSM) [2]. GSM is an all-encompassing term that underlines the multitude of genital, sexual, and urinary symptoms associated with the collection of anatomical and functional changes in vulvovaginal tissues occurring with menopause and aging, mainly the aforementioned decrease in estrogen and other sex steroids [3].

Nearly half of women who have undergone menopause have vaginal discomfort that can be attributed to VVA [1,4,5]. Menopausal women with VVA experience symptoms such as vaginal dryness, itching, burning, irritation, discharge, or bleeding, as well as dyspareunia, with lack of lubrication, discomfort, or pain [6,7]. A significant number of postmenopausal women experience progressive and chronic VVA symptoms that greatly diminish their quality of life, which is particularly pronounced in sexually active women [8]. When disclosing these symptoms to their physician, women are rather redundant, as only 25% of patients with symptoms of VVA seek medical attention [9]. Generally, women are poorly aware that VVA is a chronic condition with a significant impact on quality of life and mainly their sexual health [4,5]. Vaginal symptoms, whether minor or severe, negatively influence activities of daily living, as well as sexual activity [10]. Maintaining sexual activity in older age is a crucial component of achieving effective aging, as numerous postmenopausal women continue to engage in sexual activity, particularly if they are in a stable relationship [11,12]. The overall state of physical, psychological, and genital well-being has a considerable impact on several aspects of sexual responsiveness following menopause. Sexual function declines as individuals age, particularly in women experiencing menopause, with frequently reported symptoms such as lower libido and pain during intercourse (dyspareunia) affecting approximately half of postmenopausal women [13,14]. 

The treatment options currently available for this condition consist of vaginally administered hormones, such as estrogens and dehydroepiandrosterone (DHEA), as well as non-hormonal alternatives like lubricants and long-acting vaginal moisturizers. Additionally, systemic hormone therapy and non-hormonal oral selective estrogen receptor modulators (SERM) such as Ospemifene can also be implemented [15] and have all received approval and recommendation by the “International Society for the Study of Vulvovaginal Disease“ [16] as well as “The North American Menopause Society” [17]. However, such therapies have some problems that include contraindications, mainly in women with breast cancer [18], particularly when there is currently inadequate data to verify the safety of vaginal estrogens, DHEA, or SERMs in this specific population [17]. Low adherence to vaginal estrogens is commonly attributed to patient discontent with the use of the vaginal route, worries regarding the potential long-term effects of estrogen usage, and the high expense of medication [19]. Therefore, the implementation of a novel, efficient, and reliable treatment is paramount. The available results indicate that vaginal laser is highly effective and safe for treating symptoms and enhancing sexual function in people with VVA [15]. 

Although most of the aforementioned studies investigated the post-laser improvement of VVA, a limited number of studies investigated the change in sexual function after this intervention. As the literature lacks coherence in terms of the proposed treatments and selected outcome measures, we aimed to assess the effects of micro-ablative fractional CO_2_ laser on the clinical symptoms of VVA, as well as concordant sexual function in postmenopausal women. Our research presents a novel contribution to the field by addressing the use of laser therapy in relation to female sexual function, which has not been previously explored in our country. Moreover, our research aims to contribute to better clinical practices and outcomes for women experiencing sexual health concerns.

## 2. Materials and Methods

### 2.1. Study Design

This pilot study was conducted between January 2022 and March 2023 at our polyclinic in Belgrade. The primary goal of this study was to evaluate sexual function after the completion of a three-cycle laser procedure by using the FSFI questionnaire. The secondary goals of the study were to assess the differences in VVA symptoms and clinical signs of vaginal atrophy between the pre-treatment status and 4 weeks after complete laser treatment.

### 2.2. Participants

This study included sexually active postmenopausal women with bothersome VVA, leading to sexual health complaints. The criteria for inclusion in the study were as follows: being sexually active within the past four weeks, having no menstruation for at least 12 months, experiencing at least one subjective symptom of VVA (such as vaginal itching, burning, dryness, or dyspareunia), and/or being diagnosed with VVA during a gynecological examination. Exclusion criteria were the use of any hormonal replacement therapy (HRT) (either systemic or local) within the prior 12 months, acute or recurrent urinary tract infections, active genital infection, previous reconstructive pelvic surgery, and suffering from any hormonal imbalance or chronic condition that could interfere with study compliance.

### 2.3. Data Collection and Fractional Micro-Ablative CO_2_ Laser Treatment

Sociodemographic and anamnestic data (age, time elapsed from the last menstruation, previous deliveries, and types of deliveries) were obtained using a general questionnaire.

#### 2.3.1. VVA Symptoms Questionnaire and Vaginal Health Index Score (VHIS) 

The severity of VVA symptoms (vaginal burning, vaginal itching, vaginal dryness and dyspareunia) was self-evaluated by study participants using a 10cm visual analogue scale (VAS), where the left extreme of the scale (number 1) indicated “absence of symptom” and the right (number 10) indicated “symptom as bad as it could be”. Before treatment and 4 weeks after the third laser treatment, the condition of the vaginal mucosa was evaluated by using the Vaginal Health Index Score (VHIS), which consists of 5 characteristics of the vaginal wall: elasticity, fluid volume, pH, epithelial integrity, and moisture. The severity of each characteristic is evaluated based on the 5-point Likert scale, ranging from 1 to 5. The VHIS has a range of scores from 5 to 25, with a cut-off point below 15. A score below 15 suggests the presence of atrophic vaginitis [20]. 

#### 2.3.2. Female Sexual Function Index (FSFI)

Sexual function was evaluated before starting the first laser application and 4 weeks after the third treatment using the Female Sexual Function Index Questionnaire. The Female Sexual Function Index (FSFI) is a published instrument assessing six domains of sexual function in women: desire, arousal, lubrication, orgasm, satisfaction, and pain, as well as a total score for sexual functioning. This instrument has shown high reliability and psychometric (as well as clinical) validity in the assessment of key dimensions of female sexual function in clinical and nonclinical samples [21]. An FSFI cut-off score of 26 and scores below are classified as Female Sexual Dysfunction [22]. 

#### 2.3.3. Fractional Micro-Ablative CO_2_ Laser Treatment 

Postmenopausal women were treated intravaginally with a fractional micro-ablative CO_2_ laser system (SmartXide2 V2 LR, Monalisa Touch; DEKA, Florence, Italy), using the following settings: dot power 35 W, dwell time 1000 μs, dot spacing 1000 μm, and smart stack parameter from 1 to 3. The vaginal probe was inserted and rotated along the vaginal canal, applying laser energy to the full length of the vagina. A complete treatment cycle included three laser applications, spaced 6 to 8 weeks apart, that all participants completed. The procedure was performed in an outpatient clinic and did not require any specific preparation or anesthesia. 

### 2.4. Ethical Consideration

This study was conducted in accordance with the International Code of Medical Ethics of the World Medical Association (Declaration of Helsinki), and written informed consent was obtained from the participants after the nature and objectives of this study were fully explained to them. This study was approved by the institution’s Ethical Committee (decision number 1/2022; approved in 16 January 2022).

### 2.5. Data Analysis

Data presented in the text and tables are reported as means ± standard deviation. The numeric variables were not normally distributed and are presented as medians with an interquartile range (IQR). Continuous variable distributions were analyzed using the Shapiro–Wilk test. The Wilcoxon signed-rank test was used to define the statistical significance of continuous indicators before/after the treatment variables that did not have a normal distribution. Spearman’s correlation analysis was employed to describe the relationship among two continuous variables. Statistical analysis was performed using IBM SPSS Statistics for Windows, version 23.0 (IBM Corp., Armonk, NY, USA). The significance level was set at *p* < 0.05. All 84 participants were included in the analysis.

## 3. Results

Our study involved 84 postmenopausal women, with a mean age of 55.2 ± 5.4 years old, with a postmenopausal period that averagely lasted for 6 ± 4.8 years. Our patients had a previous delivery in 72 (85.7%) cases, out of which 60 (71.4%) were vaginal deliveries and 12 (14.3%) were deliveries by Cesarean section. As presented in Table 1, the age of our patients and the length of their postmenopausal period exhibited a significant negative correlation with VHSI scores. Specifically, older age and a longer postmenopausal period were associated with lower VHSI scores and a deterioration in vaginal health. Additionally, a longer postmenopausal period was associated with an increased severity of vaginal dryness and dyspareunia. There was no substantial association between other symptoms and either advanced age or the postmenopausal period.

Based on the VHSI cut-off score, baseline VHSI values showed that 55 (65%) of patients had VHSI scores lower than 15, indicating highly prevalent atrophic vaginitis. Prior to laser treatment, the majority of our patients displayed varying levels of symptoms related to vulvovaginal atrophy (VVA). Specifically, 70.2% of the patients experienced vaginal itching, 73.8% reported vaginal burning, 95.3% had vaginal dryness, and 86.1% had dyspareunia.

As shown in Table 2, VHSI and patient-reported VVA symptoms were significantly less pronounced after treatment with CO_2_ laser (*p* < 0.001). It is important to mention that the symptoms of vulvovaginal atrophy (VVA) either totally subsided (particularly vaginal itching and burning, with a median severity of 1, indicating the absence of symptoms) or were minimally present (VAS scores of 2 and 3 for vaginal dryness and dyspareunia).

After treatment, only one patient (1.2%) had persistent VVA, with a VHSI score of 14, with all other subjects regaining non-atrophic VHSI values of 15 or higher.

VHIS and VVA symptoms showed a significant correlation with most of the FSFI domain scores. These correlations were mainly moderate to high (Table 3). Notice that the VHIS correlates positively with the FSFI total and domain scores, indicating that a higher VHIS is associated with higher FSFI domain scores. However, the VVA symptom intensity correlation coefficients were negative, indicating an inverse relationship in which more pronounced symptoms were associated with lower FSFI scores. Vulvovaginal itching and burning severity had no significant correlation to FSFI “Orgasm” domain scores, while Itching intensity showed no significant correlation to “Arousal” domain scores as well. The severity of itching showed a strong, albeit generally mild-to-moderate, connection with other FSFI domains and total scores.

As shown in Table 4, the FSFI total and domain scores were significantly higher after treatment, indicating better sexual function, with a total score median of 26.0, a clinically relevant level as compared to baseline median total score values (18.7).

**Figure 1:**
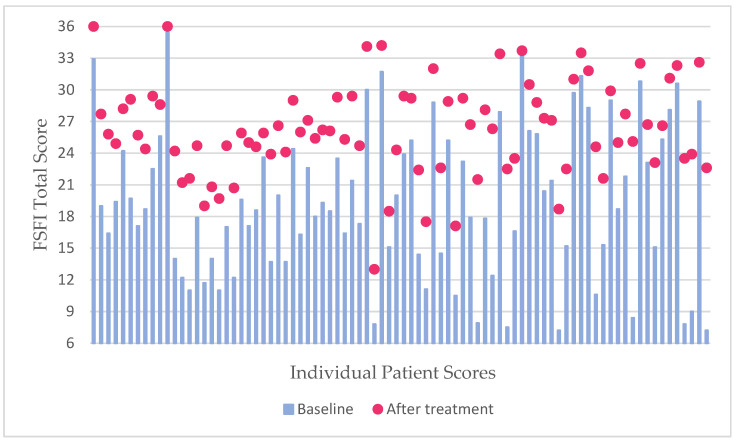
Diagram showing the baseline FSFI Total scores with a follow-up Total score after fractional CO_2_ laser treatment. Note the high peaks (values of 27 and above) after the aforementioned intervention.

The diagram (Figure 1) shows that the baseline FSFI Total scores were overall low for the majority of patients, with 69 (82.1%) cases having a score of 26 or less, indicating a high prevalence of sexual dysfunction, whereas peaks for the FSFI Total score after laser treatment were 26 and lower in around half of the cases (51.2%), showing a reduction in sexual dysfunction prevalence in our subjects, as well as significant improvement in sexual activity scores.

## 4. Discussion

Before the CO_2_ laser sessions, our cohort exhibited a high prevalence of atrophic vaginitis and pronounced VVA symptoms. Lower VHIS values and more severe VVA symptoms were correlated with lower FSFI total and domain scores. Post-treatment, VHIS, and patient-reported VVA symptoms improved significantly, leading to higher FSFI scores without reported adverse effects.

The mechanism of action of the laser consists of stimulating the gradual formation of new collagen and activating fibroblasts, resulting in the development of new trabecular-type collagen without causing harm to the surrounding tissue [23]. Vaginal tissue undergoes significant microscopical, ultrastructural, and biochemical alterations after just one hour following CO_2_ laser treatment. Specifically, the activation of regenerative mechanisms occurs in the connective tissue. This leads to the formation of new blood vessels penetrating them, as new thin fibrils of collagen III are produced in the epithelium. Fibroblasts in an active state produce new components of the extracellular matrix, such as collagen and extrafibrillar matrix molecules. This leads to an increase in the mechanical support for connective tissue and promotes the growth and maturation of epithelial cells [24]. Laser treatment effectively restores the thick squamous stratified vaginal epithelium in these settings [25]. The substantial alterations in inflammatory and modulatory cytokine patterns indicate a notable remodeling process in the vaginal epithelium, resulting in a decrease in inflammation [26]. These early-initiated regeneration mechanisms are augmented and subsequently stabilized with three laser administrations [24]. Finally, laser treatment results in a renewed and rejuvenated mucosa followed by striking clinical relief from symptoms experienced by the patients before treatment [24,25] without showing any adverse effects [23,25,26]. The histological benefits indicated above are evident in clinical practice since the fractional micro-ablative CO_2_ laser has been found to be effective and safe in treating VVA, as patients experience much-improved symptoms after treatment [27,28,29,30]. 

Before the application of CO_2_ laser treatment, our study found that 65% of patients had a VHIS lower than 15, indicating a high prevalence of atrophic vaginitis. Similar to our findings, the Italian AGATA study reported that a clinical diagnosis of VVA through routine gynecological examination displayed a prevalence ranging from 64.7 to 84.2%, occurring 1 to 6 years after menopause [31]. In our cohort, VHIS was significantly higher post-treatment, regaining non-atrophic values in 98.8% of the patients. A clinically significant increase in VHIS has been reported in other studies, also in the range of non-atrophic values [26,32,33,34,35], notable even a year after treatment [34]. When it comes to the number of laser sessions, the efficacy of 3, 4 or 5 CO_2_-laser sessions was estimated to be rather similar, as VHIS regained non-atrophic values in 80%, 96%, and 100% of the participants [35]. 

The most pronounced VVA symptoms in our subjects were vaginal dryness and dyspareunia, as these symptoms tend to be reported as most bothersome [26,36]. After the completion of laser treatment, all VVA symptoms were significantly less pronounced in our cohort, as reported by other authors as well [18,29,33,34,35,36,37,38], persisting at 12-month follow-ups [33,34]. The biggest alleviation of symptoms was that of dryness and dyspareunia, as their post-treatment levels had an intensity median of 1–2, indicating that our patients would hardly experience them after the intervention. Two systematic reviews and meta-analyses by Filippini et al. [39] and Pitsouni et al. [40] revealed that both dryness and dyspareunia were VVA symptoms that had the overall biggest reduction after micro-ablative CO_2_ laser treatment, followed by itching and burning. Similar effects of laser treatment on VVA symptom intensity were reported by Kiesel et al. [36], who concluded that fractional CO_2_ laser is a valuable therapeutic option for reducing VVA-associated genital discomfort in postmenopausal women. Several cohort studies have shown the efficacy and safety of a fractional micro-ablative CO_2_ laser for the treatment of VVA, with significantly improved symptoms post-treatment [27,28,29,30]. Namely, Salvatore et al. [29] showed that fractional CO_2_ laser treatment was effective in improving VVA symptoms, mainly vaginal dryness, dyspareunia, and vaginal itching, at the 12-week follow-up. Rosner-Tenerowicz et al. [34] reported similar results that were maintained at a 12-month follow-up, indicating laser as a therapeutic option with long-term effects. There could be certain discrepancies when it comes to the number of laser sessions needed for adequate, long-lasting results. In a study by Athanasiou et al. [35], from the third, fourth, and fifth laser sessions, dyspareunia completely regressed in 27%, 58%, and 81% of participants, dryness completely regressed in 36%, 66%, and 86%, while normal sexual function resumed in 41%, 69%, and 84% of cases, hinting a dose-response correlation, where additional sessions may further increase the rate of symptom regression.

The aforementioned results are of high importance, as vaginal dryness and dyspareunia are the most common VAA (GSM) symptoms that affect female postmenopausal sexual activity [41]. Sexual function worsens with advancing age, along with longer menopause status; the most frequently reported symptoms include low sexual desire and poor lubrication [14], as well as a lack of arousal, reduction of sexual pleasure, and satisfaction [13]. After evaluation of sexual function in our patients, our results revealed that VVA symptoms were significantly and negatively correlated with the FSFI total and domain scores. It has also been previously noted by Pinkerton et al. [42] that VVA symptoms show an approximately linear relationship with sexual functioning. A meta-analysis by Filippini et al. [39] demonstrated a significant improvement in FSFI scores following CO_2_ laser treatment, with a pooled mean difference of 11 for overall FSFI scores, bringing the clinical implications of this improvement under consideration. As the total FSFI score in our study reached a median of 26 post-treatment, with half of the cases reaching non-dysfunction total FSFI score values, it can be considered that our study achieved a clinically significant improvement in similar proportion as a three-session CO_2_ laser treatment in other studies [35]. Additionally, research has shown that normal sexual function resumes in 69% and 84% of cases for four and five laser sessions [35]; it can be assumed that more laser applications could achieve better results when it comes to sexual activity. 

Our results revealed a significant improvement in all FSFI domain scores, as reported in other studies [18,28,37], which persisted after a one-year follow-up [37]. A randomized control trial (RCT) showed that laser treatment alone brings significant improvement in dyspareunia, burning, and dryness compared to using local estrogen therapy. Utilizing laser treatment showed significant improvement in total FSFI score and individual domains of desire, as well as lubrication. However, these patients showed significant worsening of pain in the FSFI domain [43]. It is crucial to closely monitor pain following laser interventions, as research indicates that pain is the most significant factor in predicting sexual functioning. Improvement in sexual functioning is most noticeable when pain during intercourse is minimized. Corresponding levels of enhancement in other symptoms of vulvovaginal atrophy (such as dryness and itching) were associated with advantageous but lesser enhancements in sexual functioning [42]. A study on 75 women revealed that laser was equally effective with or without local estrogens when it comes to VHIS, VVA symptoms as well as FSFI scores, but the improvement in FSFI “Lubrication” domain was significant only when laser with adjuvant moisturizers was utilized [44]. 

Although hormonal treatment may be currently considered the “gold standard”, laser treatment has the potential to completely regress the VVA symptoms with the concordant reestablishment of normal sexual function in postmenopausal women. Furthermore, considering the previously noted low compliance for local estrogens, which is owing to its low efficacy, potential risks associated with long-term estrogen usage, or their high cost [19], laser therapy appears to be a viable option for treating VVA in menopausal women, as it has shown to be successful as a standalone treatment [44]. Moreover, the use of a CO_2_ laser demonstrated a favorable safety profile with no significant negative outcomes identified [39]. Given the inconsistency of data on the long-term effects of laser treatment, the “International Society for the Study of Vulvovaginal Disease” does not endorse the use of laser treatment outside the setting of clinical trials [16]. “The North American Menopause Society” accents that there are insufficient placebo-controlled trials of energy-based therapies like laser, to draw conclusions on efficacy and safety or to make treatment recommendations [17]. Hence, further high-quality research, specifically randomized control trials, is needed to determine which specific pathologies can be treated, their long-term efficacy and other effects, if maintenance treatment is necessary, and long-term safety concerns. Moreover, CO_2_ laser treatment is highly costly and a practice that remains relatively uncommon among gynecologists, thus potentially restricting its accessibility [45].

While our findings demonstrate considerable potential for future clinical utilization of the CO_2_ laser in treating menopausal VVA and sexual dysfunction, this pilot study has several limitations. Firstly, this was a single-center, non-randomized study with no control group (placebo or other treatment types). Our cohort was rather small, and we lacked long-term follow-up. We used self-reported instruments for assessing VVA symptomatology, as well as sexual functioning. Conversely, our study was primarily designed to showcase the practicality and effectiveness of using laser treatment in the vaginal regions of postmenopausal women. We believe this is a strength of this study to guide further research in populations of women with contraindications to hormonal treatments.

## 5. Conclusions

Our study revealed that a three-cycle fractional CO_2_ laser treatment is a useful treatment option to alleviate VVA-associated genital discomfort, particularly dyspareunia and vaginal dryness in postmenopausal women. Furthermore, it improved overall vaginal health by elevating the VHIS scores to non-atrophic levels. The resulting improvements were subsequently accompanied by a rise in the sexual functioning scores. While these findings demonstrate significant potential for future clinical application, additional research is needed to assess its long-term effectiveness, potential side effects, and safety concerns.

## Figures and Tables

**Table 1:** Association of age and postmenopausal period duration with vulvovaginal atrophy symptoms severity.

	VHSI	Vaginal Itching	Vaginal Burning	Vaginal Dryness	Dyspareunia
Age (years)	−0.257 *	0.033	0.106	0.083	0.159
Postmenopause duration (years)	−0.431 **	0.192	0.185	0.283 **	0.312 **

VHSI—Vaginal Health Index Score; Values are presented as Spearman correlation coefficients; * *p* < 0.05; ** *p* < 0.001.

**Table 2:** Vulvovaginal atrophy symptom severity at baseline level and after treatment with CO_2_ laser.

	Baseline	After Treatment	*p* Value **
VHSI	13 (12–15.5)	19 (17–21)	<0.001
Vaginal Itching *	2 (1–4)	1 (1–2)	<0.001
Vaginal Burning *	3 (1.5–4)	1 (1–2)	<0.001
Vaginal Dryness *	5 (3–8)	2 (1–3)	<0.001
Dyspareunia *	5 (2–8)	2 (1–3)	<0.001

VHSI—Vaginal Health Index Score; Values are presented as median (interquartile range); * Measured on a 10-cm VAS scale (range 0–10) **; Wilcoxon Signed Ranks Test, significant at *p* < 0.05.

**Table 3:** Correlation coefficients.

	FSFI Scores						
	Desire	Arousal	Lubrication	Orgasm	Satisfaction	Pain	Total
VHSI	0.456 **	0.463 **	0.595 **	0.446 **	0.424 **	0.600 **	0.599 **
Vaginal itching	−0.285 *	−0.195	−0.305 *	−0.148	−0.238 *	−0.319 *	−0.305 *
Vaginal burning	−0.356 *	−0.234 *	−0.477 **	−0.163	−0.310 *	−0.474 **	−0.414 **
Vaginal dryness	−0.414 **	−0.392 **	−0.660 **	−0.385 **	−0.347 **	−0.646 **	−0.590 **
Dyspareunia	−0.500 **	−0.499 **	−0.667 **	−0.470 **	−0.382 **	−0.768 **	−0.692 **

VHSI—Vaginal Health Index Score; Values are presented as Spearman correlation coefficients; * *p* < 0.05; ** *p* < 0.001.

**Table 4:** FSFI domain scores before and after treatment with CO2 laser.

	Before Treatment	After Treatment	*p* Value *
Total	18.7 (14.2–24.8)	26.0 (23.7–29.2)	<0.001
Desire	3.6 (2.4–3.6)	4.2 (3.6–4.8)	<0.001
Arousal	3.0 (2–4.2)	4.5 (3.9–4.8)	<0.001
Lubrication	3.0 (1.8–4.2)	4.2 (3.9–4.8)	<0.001
Orgasm	3.2 (2.4–4.4)	4.4 (4–5.2)	<0.001
Satisfaction	3.6 (2.4–4.4)	4.4 (3.6–5)	<0.001
Pain	3.2 (1.6–4.4)	4.4 (3.6–5.2)	<0.001

Values are presented as median (interquartile range); * Wilcoxon signed-rank test, significant at *p* < 0.05.

## Data Availability

The data presented in this study are available upon request from the corresponding author.
